# Impact of lactate on immune cell function in the tumor microenvironment: mechanisms and therapeutic perspectives

**DOI:** 10.3389/fimmu.2025.1563303

**Published:** 2025-03-26

**Authors:** Xuan-Yu Gu, Jia-Li Yang, Rui Lai, Zheng-Jun Zhou, Dan Tang, Long Hu, Li-Jin Zhao

**Affiliations:** ^1^ Department of General Surgery, Digestive Disease Hospital, Affiliated Hospital of Zunyi Medical University, Zunyi, China; ^2^ Department of Otolaryngology-Head and Neck Surgery, Affiliated Hospital of Zunyi Medical University, Zunyi, China; ^3^ Hepatobiliary and Pancreatic Surgery, the Second Affiliated Hospital of Zunyi Medical University, Zunyi, China; ^4^ Department of Hepatobiliary and Pancreatic Surgery, Suzhou Medical College of Soochow University, Suzhou, China; ^5^ Wisdom Lake Academy of Pharmacy, Xi’an Jiaotong-Liverpool University, Suzhou, China

**Keywords:** lactate metabolism, tumor microenvironment, immunosuppression, targeted therapy, immunotherapy

## Abstract

Lactate has emerged as a key regulator in the tumor microenvironment (TME), influencing both tumor progression and immune dynamics. As a byproduct of aerobic glycolysis, lactate satisfies the metabolic needs of proliferating tumor cells while reshaping the TME to facilitate immune evasion. Elevated lactate levels inhibit effector immune cells such as CD8^+^ T and natural killer cells, while supporting immunosuppressive cells, such as regulatory T cells and myeloid-derived suppressor cells, thus fostering an immunosuppressive environment. Lactate promotes epigenetic reprogramming, stabilizes hypoxia-inducible factor-1α, and activates nuclear factor kappa B, leading to further immunological dysfunction. In this review, we examined the role of lactate in metabolic reprogramming, immune suppression, and treatment resistance. We also discuss promising therapeutic strategies targeting lactate metabolism, including lactate dehydrogenase inhibitors, monocarboxylate transporter inhibitors, and TME neutralization methods, all of which can restore immune function and enhance immunotherapy outcomes. By highlighting recent advances, this review provides a theoretical foundation for integrating lactate-targeted therapies into clinical practice. We also highlight the potential synergy between these therapies and current immunotherapeutic strategies, providing new avenues for addressing TME-related challenges and improving outcomes for patients with cancer.

## 1 Introduction

Lactate, a byproduct of glycolysis, plays a critical role in shaping the tumor microenvironment (TME). Under normal conditions, pyruvate from glycolysis enters the mitochondria to undergo oxidative phosphorylation (OXPHOS), generating 36 adenosine triphosphates (ATP) per glucose molecule. However, in the context of tumorigenesis, tumor cells shift to aerobic glycolysis, known as the Warburg effect, in which pyruvate is converted to lactate by lactate dehydrogenase A (LDHA) even in the presence of oxygen (1). This metabolic adaptation allows tumors to sustain rapid proliferation by maintaining glycolytic flux despite a lower ATP yield (2 ATP per glucose molecule). In addition to glucose metabolism, glutamine metabolism provides an alternative source of lactate, which further fuels tumor growth.

Lactate accumulation in tumors is regulated by hypoxia-inducible factor-1α (HIF-1α) and c-Myc. These factors enhance the expression of glycolytic enzymes such as hexokinase 2 (HK2) and glucose transporter 1 (GLUT1) while inhibiting pyruvate dehydrogenase (PDH), which normally channels pyruvate into the mitochondrial tricarboxylic acid (TCA) cycle (2). As a result, lactate accumulates within tumor cells, indicating the need for efficient export mechanisms to prevent intracellular acidification and maintain metabolic homeostasis.

Monocarboxylate transporters (MCTs) mediate lactate transport in the TME. MCT4, which is highly expressed in glycolytic tumor cells, facilitates lactate and proton (H^+^) export, leading to extracellular acidification. The acidic environment promotes tumor invasion, angiogenesis, and immune suppression (3). In contrast, MCT1 supports bidirectional lactate transport, enabling oxidative tumor cells and cancer-associated fibroblasts (CAFs) to absorb lactate as energy sources (4). This metabolic cooperation sustains tumor progression by allowing different cell populations to adapt to changing nutrient and oxygen conditions.

The acidification of the TME has profound effects on immune function. A low extracellular pH directly impairs cytotoxic T-cell and natural killer (NK) cell activity, reducing their ability to kill tumor cells (5). At the same time, lactate stabilizes HIF-1α and activates NF-κB, promoting an immunosuppressive phenotype (6). This change in the acidic environment enhances the function of regulatory T cells (Tregs) and myeloid-derived suppressor cells (MDSCs) while inducing tumor-associated macrophages (TAMs) to adopt an M2-like phenotype, further dampening antitumor immunity (7). These metabolic and immune adaptations not only drive tumor progression but also contribute to resistance to immune checkpoint inhibitors (ICIs).

Lactylation is a recently discovered post-translational modification (PTM) that was originally described by Zhao et al. in 2019 (8). Increased lactate concentrations enhance lactylation via multiple pathways, subsequently affecting cellular functions and disease progression (9). In this process, lactate coenzyme A (L-lactyl-CoA) covalently modifies lysine residues (10). “Writer” enzymes such as p300/CBP mediate lactylation, whereas “eraser” enzymes such as HDAC1-3 and SIRT1-3 regulate its reversibility (11). Lactylation occurs in both histone and non-histone proteins and is closely linked to cellular metabolic activity. Lactylation significantly affects chromatin remodeling, gene expression, and cellular function in the TME (12).

Given its central role in tumor metabolism and immune suppression, lactate metabolism and transport, as well as lactylation, are promising targets for cancer therapy. Strategies such as blocking MCTs, inhibiting lactate production, or modulating TME acidity could enhance immune cell function and improve the efficacy of existing cancer therapies. Understanding the interplay between lactate metabolism, lactylation, and immune evasion is essential for developing new interventions that restore antitumor immunity and improve patient outcomes. In this review, we explore the complex role of lactate in tumor progression, its impact on immune regulation, and potential therapeutic strategies to counteract lactate-driven immunosuppression.

## 2 Role of lactate in the TME and immunotherapeutic strategies

### 2.1 Immunoregulatory role of lactate in the TME: intersection of immunosuppression and metabolic reprogramming

**Figure 1:**
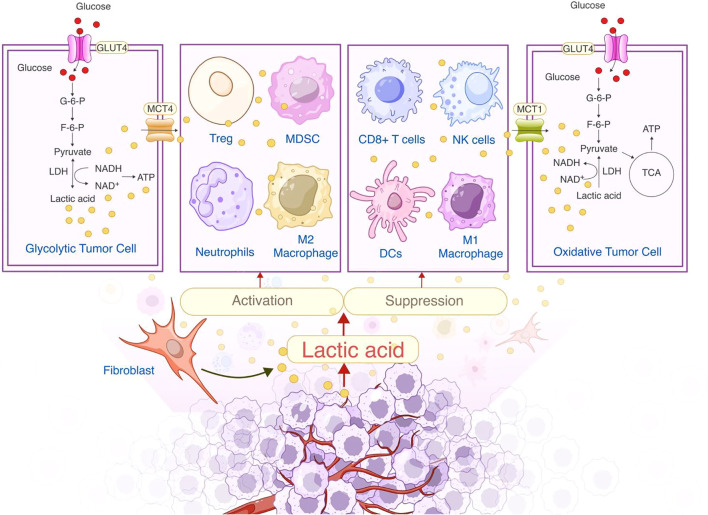
Dual role of lactate in the tumor microenvironment: Metabolic regulation and immunosuppression.

As outlined in the previous section, lactate metabolism and transport lead to its accumulation in the TME, where its concentration can reach 20–40 mM, compared to 1.5–3 mM in normal tissues (13). This accumulation significantly lowers the extracellular pH to 6.0–6.5, well below the normal range of 7.3–7.4. Such an acidic environment impairs immune cell function and metabolism, promoting tumor immune evasion—a hallmark of cancer development (14, 15).The acidic pH reduces the metabolic flexibility of CD8^+^ T cells and NK cells while enhancing the immunosuppressive properties of Tregs and MDSCs. The role of lactate in the TME is illustrated in **Figure 1**.

Different cancer types exhibit varying degrees of lactate accumulation (**Table 1**). For example, lactate concentrations are significantly higher in tumors with metastatic spread (12.3 ± 3.3 μmol/g) than in those without metastasis (4.7 ± 1.5 μmol/g) (16). In contrast, cervical cancer samples show a more modest increase in lactate, with concentrations of 10.0 ± 2.9 μmol/g in tumors with metastasis and 6.3 ± 2.8 μmol/g in those without (13). In breast cancer, lactate concentrations vary widely, with values of 0.6–8.0 μmol/g in late-stage tumors (17). The variations in lactate concentration indicate that distinct tumor types exhibit differences in lactate accumulation, potentially influencing their vulnerability to metabolically targeted therapies. Lactate promotes immunosuppression and tumor progression, but its role in the TME of different tumor types is unclear. Therefore, an in-depth study of the function of lactate in the TME will help to understand its role in tumor metabolism and provide ideas for future targeted therapies.

**Table 1:** Lactate concentration and metastatic spread in cancer types.

Cancer Type	Lactate Concentration	References
Head and Neck Cancer	12.3 ± 3.3 μmol/g (with spread) 4.7 ± 1.5 μmol/g (without spread)	(16)
Colorectal Cancer	13.4 ± 3.8 μmol/g (with spread) 6.9 μmol/g (without spread)	(154)
Breast Cancer	0.6–8.0 μmol/g (median concentration range)	(17)
Cervical Cancer	10.0 ± 2.9 μmol/g (with spread) 6.3 ± 2.8 μmol/g (without spread)	(13)
Lung Cancer (Metastatic)	1.8 ± 2.2 mmol/L (maximal levels)	(155)
Astrocytomas	12.35 mmol/L (with spread) 8.28 mmol/L (without spread)	(156)

Metabolic reprogramming is the adjustment of cellular metabolic pathways and product distribution in response to physiological or pathological conditions, enabling cells to adapt to growth, differentiation, and environmental changes. In the tumor microenvironment, this process alters immune cell function by modifying metabolic substrates and signaling pathways (18). These connections between lactate metabolism and immune suppression highlight the pivotal role of metabolic reprogramming in shaping tumor immune environments. In the subsequent sections, we explore the varied effects of lactate accumulation on different immune cell types and the implications of lactate accumulation for therapeutic strategies aimed at countering immune evasion.

#### 2.1.1 Inhibitory effects of lactate on effector immune cells

##### 2.1.1.1 T lymphocytes

Lactate is a key regulator that inhibits T-cell antitumor activity at multiple levels, including by suppressing T-cell receptor signaling by decreasing p38 and JNK phosphorylation, which lowers the production of interferon-γ (IFN-γ) and tumor necrosis factor-α (19–21), thereby inhibiting T-cell proliferation and cytotoxicity. Lactate also affects the NAD^+^/NADH redox balance, inhibiting glycolysis, decreasing ATP production, and contributing to T-cell exhaustion (19, 22). In high-lactate environments, such as those found in melanoma and other tumor models, NAD^+^ depletion leads to T-cell apoptosis and a significant reduction in antitumor responses (23, 24).

Interestingly, recent studies have identified lactate as a TCA fuel for certain tumor cells and untransformed tissues *in vivo* (25–27). It is an important carbon source for CD8^+^ T cells, and at its physiological concentration, lactate promotes mitochondrial oxidation and supports OXPHOS, thereby enhancing energy production and cellular biosynthesis, especially during functional T cell responses *in vivo* (28). Similarly, lactate produced by innate immune cells or tumor cells may provide energy for effector T cells in inflammatory tissues (29, 30). Lactate serves a dual function in T-cell immunity, seemingly contingent upon varying lactate concentrations. Lactate enhances T-cell metabolism and activity at lower doses (0.5–3 mmol). However, at concentrations exceeding 5 mmol, lactate impedes T cell functionality and results in T cell dysfunction.

Furthermore, lactate plays a direct role in driving T-cell exhaustion through its influence on immune checkpoint pathways. Lactic acid enhances the expression of programmed death ligand 1 (PD-L1) on tumor cells through its receptor GPR81, which inhibits CD8+ T cell-mediated cytotoxicity and fosters immune tolerance in the TME (31, 32). Additionally, lactate-driven SIRT1 activation results in deacetylation and degradation of T-bet, a transcription factor essential for maintaining CD8^+^ T cell effector function, thereby shifting T cells toward a more exhausted phenotype with higher PD-1 expression and diminished cytokine production (33).

Lactate also modulates CTLA-4 signaling, further reinforcing T cell dysfunction. Increased extracellular lactate enhances FOXP3 expression in Tregs, promoting an immunosuppressive microenvironment that inhibits the activation of effector T cells via CTLA-4–mediated suppression (34). Moreover, lactate influences T cell dysfunction by modulating chemokine production and receptor signaling, thereby restricting T cell infiltration into tumor tissues (35, 36). This phenomenon not only prevents effective immune surveillance but also intensifies the PD-1/CTLA-4-driven dysfunction state by further inhibiting T cell function in proinflammatory circuits that could otherwise restore function.

These inhibitory effects significantly impair immune cell function in the TME, facilitating tumor immune evasion. Given the profound role of lactate in sustaining T-cell exhaustion via the PD-1/CTLA-4 axis, therapeutic strategies targeting lactate metabolism, such as MCT inhibitors, LDHA blockade, or buffering agents, may enhance the efficacy of ICIs by reprogramming dysfunctional T cells and restoring their antitumor activity. Future studies should further investigate how lactate-targeting approaches could synergize with ICIs to reverse exhaustion and reinvigorate T-cell function within the TME.

##### 2.1.1.2 NK cells

Lactate diminishes the antitumor efficacy of NK cells in the TME via various pathways, including direct metabolic effects, immune modulation, and acidic environments. Elevated lactate concentrations (> 15 mM) suppress the NFAT signaling pathway in NK cells, reducing IFN-γ production and impairing antitumor signaling (21, 37). Lactate-induced intracellular acidification disrupts lipid biosynthesis, impairs mitochondrial function, and increases oxidative stress, ultimately resulting in NK cell apoptosis (38). These metabolic disturbances significantly reduce NK cell cytotoxicity and overall antitumor capacity. Lactate also suppresses critical cytokine synthesis in NK cells by inhibiting the mTOR signaling pathway, which is essential for their activation and function (38, 39).

Furthermore, lactate inhibits NK cell activity, primarily by acidifying the TME, which impairs NK cell degranulation and reduces key cytotoxic markers such as perforin and CD107a in various cancer models, including pancreatic and breast cancers (40–42). Therapeutic strategies that neutralize acidity and target lactate metabolism show promise in reversing these immunosuppressive effects.

##### 2.1.1.3 Dysfunction of Dendritic Cells (DCs)

Lactate inhibits DC activity in the TME through multiple mechanisms, significantly impairing their antitumor immune functions. This suppression is primarily due to the interference of lactate with DC development and maturation, which prevents DCs from effectively presenting antigens and activating effector T cells. Lactate inhibits the expression of MHC-II and co-stimulatory molecules such as CD80 and CD86, leading to DC dysfunction (43, 44). Lactate also stimulates IL-10 secretion while inhibiting IL-12 synthesis, further diminishing the ability of DCs to activate cytotoxic T lymphocytes (CTLs) (45, 46). In melanoma models, inhibiting lactate production enhances DC function and antitumor efficacy, highlighting the fundamental role that lactate plays in DC dysfunction (47).

Glycolysis is a key metabolic process in DCs for antigen presentation and adaptive immune responses. However, lactate disrupts DC energy supply by inhibiting glycolysis and the calcium signaling pathway by activating GPR81, thereby worsening intracellular acidification and ultimately impairing DC function (48, 49). It has also been demonstrated that DC motility and migration are largely dependent on glycolysis (50), and thus, lactate-induced glycolysis impairs the ability of DCs to mount antitumor immunity. Lactate also activates SREBP2 in tumor DCs, driving the conversion of conventional DCs into mature regulatory DCs, which are then directed to tumor-draining lymph nodes, thereby inhibiting DC-mediated antigen cross-presentation (51).

#### 2.1.2 Enhancing effect of lactate on immunosuppressive cells

##### 2.1.2.1 Regulatory T cells

Lactate in the TME significantly affects the metabolic balance, signaling pathways, and epigenetic regulation of Tregs, enhancing their immunosuppressive capabilities and contributing to tumor immune evasion (52). Studies have shown that elevated MCT1 expression enables Tregs to absorb and utilize lactate more efficiently, enhancing their survival and functionality (53). In mouse melanoma models, Tregs lacking MCT1 exhibit decreased lactate uptake, reduced immunosuppressive abilities, and diminished tumor-promoting actions, highlighting the pivotal role of lactate in Treg regulation (27, 54, 55).

In addition to metabolic adaptation, lactate influences Tregs through signaling pathways and epigenetic modifications that enhance their stability and suppressive function. Lactate activates the PI3K/Akt/mTOR pathway, increasing FOXP3 expression and enhancing Treg-mediated suppression of effector T cells (56). Lactate also promotes Treg recruitment and infiltration into the TME by upregulating chemokines such as CXCL12 and CX3CL1, further contributing to an immunosuppressive environment (55, 57).

Lactate also enhances Treg immunosuppression by boosting the release of cytokines such as IL-10 and TGF-β (58, 59), which intensify the inhibitory effects of Tregs on effector immune cells, facilitating immune evasion. Comparative studies have shown that lactate-rich environments foster greater Treg suppressive activity than glucose-rich environments, highlighting the crucial role of lactate in Treg-mediated immunosuppression (52).

##### 2.1.2.2 Myeloid-derived suppressor cells

In addition to enhancing Treg-mediated immunosuppression, lactate drives the activity and recruitment of MDSCs, a key component of the immunosuppressive network in the TME. Lactate directly promotes the metabolic adaptation and immunosuppressive function of MDSCs through complex control involving epigenetic modifications and signaling networks. Lactate affects MDSC metabolism by modulating HIF-1α activity, promoting the upregulation of ARG1 and iNOS expression (60, 61). This depletes arginine, which is essential for T-cell activation, while producing reactive oxygen species and nitric oxide, which directly impair the functions of effector T and NK cells (62–64). Furthermore, lactate improves the immunosuppressive potential of MDSCs by upregulating PD-L1 expression (60, 65). In breast cancer models, lactate-induced HIF-1α activation enables MDSCs to suppress effector T cells and accelerate tumor growth (66).

Beyond metabolic control, lactate boosts MDSC recruitment and accumulation in the TME by acting as a chemoattractant and activating the mTOR and GPR81 signaling pathways (67, 68). A pancreatic cancer study showed that lactate-mediated GPR81 activation significantly promoted MDSC growth and immunosuppressive activity (7, 60, 69). Furthermore, lactate indirectly inhibits NK cell activity by promoting the growth and activation of MDSCs (7, 70).

##### 2.1.2.3 Tumor-associated macrophages

TAMs undergo significant metabolic and gene expression changes during lactate-driven reprogramming, typically polarizing toward the immunosuppressive M2 phenotype (71). Lactate enhances the immunosuppressive function of TAMs by activating the ERK/STAT3 and GPR132 signaling pathways, in part through its regulation of HIF-1α (72, 73). Activation of these pathways boosts the production of vascular endothelial growth factor (VEGF) and ARG1, promoting angiogenesis and immune evasion (74). Lactate also encourages M2-polarized TAMs to release immunosuppressive molecules (IL-10 and TGF-β), which impair the activity of CTLs and NK cells while recruiting Tregs (71, 75–77). Furthermore, lactate-driven metabolic reprogramming shifts the energy supply of TAMs from glycolysis to OXPHOS, making them more adaptable to the hypoxic, high-lactate TME (78, 79). This metabolic transition, in which lactic acid is efficiently converted to pyruvate, is mainly due to the preferential dependence of M2 macrophages on mitochondrial metabolism (80–82). Pyruvate then enters the mitochondria via the MPC1 transporter and enters the TCA cycle, further supporting OXPHOS (83). This process ensures that M2 macrophages are able to receive continuous energy production. This adaptation increases the release of matrix-degrading enzymes, such as matrix metalloproteinases, which promote extracellular matrix remodeling, tumor invasion, and metastasis (79, 84).

These lactate-induced changes in TAM function have been observed in various cancer models, including breast and gastric cancer and melanoma, highlighting their crucial role in tumor development, immune evasion, and resistance to treatment (85). These findings highlight the importance of TAMs in the TME and indicate that targeting lactate metabolism and TAM polarization may offer promising strategies for antitumor therapies.

#### 2.1.3 Effects of lactylation on TME immunoregulation

##### 2.1.3.1 Lactylation: an emerging PTM and its biological functions

Lactylation has been newly discovered and plays a crucial role in immune regulation through transcriptional activation. For example, H3K18la lactylation on histone 3 enhances chromatin accessibility and activates the transcription of tumor-promoting genes (86). This modification upregulates arginase-1 (ARG1) and interleukin (IL)-10 in TAMs, driving them toward an immunosuppressive M2 phenotype and facilitating tumor immune escape (73). In addition to histones, lactylation affects non-histone proteins, leading to various physiological effects. For example, lactylation of Aldolase A inhibits glycolysis, and modification of RIG-I suppresses the NF-κB signaling pathway, maintaining an immunosuppressive TME (87).

In addition to immune cells, lactylation may regulate key stromal components of the TME, including cancer-associated fibroblasts (CAFs) and endothelial cells. CAFs play a critical role in extracellular matrix remodeling, metabolic crosstalk, and immune modulation, and accumulating evidence suggests that lactate metabolism may influence their activation (88). Recent studies have indicated that lactylation of histone marks may enhance the transcription of pro-tumorigenic genes, such as fibroblast activation protein (FAP) and α-SMA (ACTA2), promoting fibroblast-mediated extracellular matrix remodeling and tumor progression (89). This effect is likely mediated through TGF-β and Notch signaling pathways, both of which have been implicated in lactylation-induced fibroblast activation (90, 91).

Similarly, endothelial cells exposed to high lactate levels exhibit increased angiogenic potential, and lactylation may contribute to the expression of angiogenesis-related genes, such as VEGFA and ANGPT2, thereby facilitating neovascularization within tumors. Mechanistically, HIF-1α lactylation enhances VEGF signaling, driving tumor vascularization even under normoxic conditions (92). However, whether lactylation directly modifies VEGF-related transcription factors or acts through broader chromatin remodeling remains unclear.

Collectively, lactylation serves as a critical link between metabolic shifts and epigenetic regulation, contributing to an immunosuppressive and tumor-supportive microenvironment. In addition to immune regulation, its emerging roles in fibroblast activation and angiogenesis suggest that lactylation-dependent pathways may serve as promising therapeutic targets. However, further research is needed to delineate the precise molecular mechanisms by which lactylation shapes tumor-stroma interactions, providing new opportunities for metabolism-based cancer therapies.

##### 2.1.3.2 Lactylation as a key epigenetic regulator of immune cell activity in the TME

**Figure 2:**
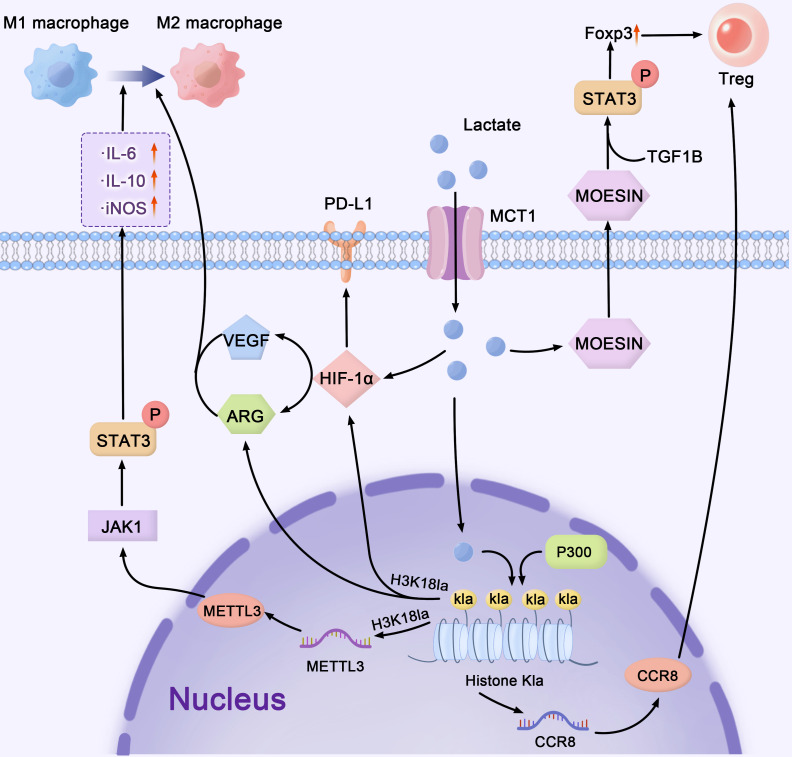
Lactate-mediated crosstalk: bridging metabolism, epigenetics, and immune regulation in the tumor microenvironment. lactate, as a major mediator, connects cellular metabolism, transcriptional control, and immunological signaling networks in the TME. Lactate enters cells through MCT1 and upregulates MOESIN, stimulating the TGF-β/STAT3 pathway to enhance Treg cell development and immunological suppression. Lactate regulates HIF-1α stability, VEGF, and PD-L1 expression, facilitating immune evasion. In the epigenetic landscape, METTL3 promotes gene transcription through m6A RNA alterations, whereas p300 acetylates histones (Kla), hence increasing transcriptional activity. These interrelated pathways govern the TME by balancing M1 and M2 macrophage polarization, as well as directing immunological homeostasis and tumor growth. The pathways shown represent select examples of lactate’s downstream effects on immune modulation. TME, tumor microenvironment; MCT, monocarboxylate transporters; VEGF, vascular endothelial growth factor; PD-L1, programmed death ligand 1.

Lactate induces lactylation of both histone and non-histone proteins, significantly altering the functional states of CD8^+^ T cells, Tregs, TAMs, MDSCs, and other immune cells (**Figure 2**). Studies have shown that lactate stimulates histone lactylation (e.g., H3K18la), which regulates the expression of MCT1 and MCT4 (93). One of the key effects of lactylation is its role in immune checkpoint regulation. This effect is partly mediated by lactate-induced histone lactylation, which upregulates PD-1 transcription and reinforces the exhausted phenotype (94). In addition, lactylation significantly affects effector immune cells such as NK and CD8^+^ T cells. In acute myeloid leukemia, lactate-induced H4K5la changes enhance PD-L1 expression, which directly inhibits CD8^+^ T cell activation (95).

The aforementioned text indicates that lactate can influence Treg function via lactylation. Lactate modulates TGF-β signaling in Tregs by lactylating MOESIN, thus stabilizing FOXP3 expression and promoting a metabolic shift from glycolysis to OXPHOS (96). Lactate-induced histone lactylation drives the transcription of genes associated with Treg activity and stabilizes FOXP3 expression through epigenetic mechanisms (96, 97). In multiple tumor models, including human colorectal cancer tissues, histone lactylation at FOXP3 regulatory regions correlates with heightened Treg activation and tumor progression (12, 98). This metabolic reprogramming enhances Treg survival in the TME and amplifies their immunosuppressive function. Lactate also regulates the expression of the chemokine receptor CCR8, facilitating Treg migration to tumors (99).

Lactylation also critically influences macrophage and MDSC activity. Lactate induces H3K18la histone modifications in the TME, driving the polarization of proinflammatory M1 macrophages toward the immunosuppressive M2 phenotype (100). Similarly, in MDSCs, lactate-induced histone lactylation increases the expression of key immunosuppressive genes. For example, in melanoma and lung cancer models, histone lactylation upregulates IL-10 production, further supporting MDSC expansion and suppressive activity (7). These synergistic processes highlight the crucial role of lactate in the promotion of MDSC-mediated immunosuppression. Lactate-induced lactylation of METTL3 regulates m6A RNA modifications, which increase STAT3 phosphorylation and promote the release of IL-6 and IL-10, further supporting immunosuppressive signaling (101).

Overall, lactylation is a key regulator of immune cell activity within the TME and plays a crucial role in tumor immune evasion through its dual influence on metabolism and epigenetics. From histone lactylation upregulating PD-1 and FOXP3 expression to METTL3 lactylation enhancing m6A RNA modifications and IL-10 production, lactate-induced epigenetic modifications orchestrate an intricate immunosuppressive network. These interconnected pathways form a critical regulatory system that drives tumor progression. The complex role of lactate in the regulation of immune cell function highlights its potential as a therapeutic target. Indeed, novel strategies could restore immune cell function and boost antitumor immunity by disrupting lactate-driven immunosuppressive networks, blocking histone lactylation, or neutralizing TME acidity.

### 2.2 Lactate metabolism and tumor immunotherapy: targeting strategies and future directions

#### 2.2.1 Neutralizing acidity in the TME: an innovative strategy for restoring immune cell function

The acidic TME resulting from lactate accumulation significantly impairs the function of CTLs and NK cells, promoting immune evasion and tumor growth. To address this issue, several novel strategies have been developed to neutralize the acidity of the TME and restore immune cell function. **Table 2** outlines potential extracellular pH (pHe) modulation techniques, including buffering agents such as sodium bicarbonate and carbonic anhydrase IX (CAIX) inhibitors.

**Table 2:** pHe modulation targets in tumor microenvironment.

Compound	Cancers	Research status
Sodium Dichloroacetate (DCA)	Breast cancer; prostate cancer; colon cancer; melanoma; glioma; acute myeloblastic leukemia; myeloma; lung cancer	Preclinical and Phase IV (147, 157–164)
Bafilomycin A1	Lung adenocarcinoma; breast cancer; glioma	Preclinical (165, 166)
Bromopyruvate	Glioma	Preclinical (167)
Esomeprazole	Glioma	Preclinical (168)
Omeprazole, Pantoprazole, Lansoprazole	Glioma	Preclinical (168–172)
Sodium Bicarbonate	Breast cancer	Preclinical (105)
Acetazolamide	Lymphoma;	Clinical Practice Research (173)
Sodium Bicarbonate	Pancreatic cancer	Phase I/II (174)
SLC-0111	Colorectal cancer; lung cancer; clear cell carcinoma of the kidney; and sarcoma	Phase I (117)
G250 (NCT04969354)	Renal cell carcinoma	Recruiting
Lonidamine (NCT00435448)	Prostate cancer	Phase III (175)

Lactate-induced immunosuppression in NK cells can be mitigated by neutralizing the acidic TME. Buffering agents, such as NaHCO₃, can raise the pHe in tumor tissues, reducing TME acidity (102). Bicarbonate buffers, which elevate pH, have demonstrated the ability to restore NK cell functions, including IFN-γ production and cytotoxicity (103, 104). In lymphoma models, these buffering strategies significantly enhance tumor control, emphasizing the therapeutic potential of lactate-targeted interventions (103, 104). Preclinical breast cancer models have shown that NaHCO₃ enhances T-cell infiltration and activation, considerably increasing the levels of cytokines such as IFN-γ, IL-2, and IL-12p40 (105). Alkalizing the TME with NaHCO₃ also reduces PD-L1 expression, enhances T cell activity, and improves ICI efficacy. This combined approach has demonstrated notable tumor regression in preclinical studies, highlighting the therapeutic potential of buffering therapy (105). However, systemic pH modulation lacks specificity, and off-target effects on normal tissues remain a concern, limiting its broader clinical application.

Another pH-modulating strategy involves proton pump inhibitors (PPIs). For instance, esomeprazole improves CTL activity and slows tumor progression by modulating the local tumor pH (106). A phase III study (NCT01069081) found that intermittent high-dose esomeprazole enhanced chemotherapy effectiveness (docetaxel and cisplatin) in patients with metastatic breast cancer while reducing toxicity (107). Furthermore, PPIs such as omeprazole exhibit radiosensitizing effects by inducing G1 phase cell-cycle arrest through p21 overexpression, impairing DNA repair and enhancing radiation efficacy (108, 109). While promising, the main benefit of PPIs may be in combination with radiation and chemotherapy. In a clinical trial combining PPIs with atezolizumab, researchers found that PPI use was a negative prognostic indicator for patients with non-small cell lung cancer (110). Similarly, another meta-analysis showed that PPIs reduced the effectiveness of ICIs (111).

In contrast to buffering agents and PPIs, CAIX inhibitors provide a tumor-specific approach to regulating pH. Overexpressed in hypoxic tumor regions, CAIX catalyzes the conversion of CO₂ to bicarbonate (HCO₃⁻) and protons (H^+^), maintaining an acidic environment that supports tumor cell survival and resistance to treatments (112–116). Inhibiting CAIX with drugs such as SLC-0111 disrupts the pH balance, increasing the susceptibility of cancer cells to chemotherapy and radiation (117). This selective targeting reduces off-target effects and is a promising approach for integration with existing therapies. However, CAIX inhibitors have significant drug delivery limitations at physiologic pH (7.4). Studies have shown that although CAIX inhibitors such as U-104 can enhance tumor cell penetration of drugs (e.g., adriamycin) in an acidic environment, they do not significantly improve drug delivery efficiency at physiological pH (118). Therefore, combination therapy may be an effective strategy to improve therapeutic efficacy.

In summary, each of these approaches offers distinct advantages and challenges. Although buffering agents effectively raise the pH of the TME, their systemic effects limit their targeted application. Moreover, PPIs, although useful in modulating tumor acidity, primarily benefit chemotherapy and radiation response rather than directly restoring immune function. CAIX inhibitors present a more precise strategy but require improved tumor-specific delivery mechanisms to overcome resistance and maximize efficacy (119). Considering these differences, combining pH modulation with other immunotherapies may provide an optimal strategy for overcoming TME-induced immune suppression. For instance, buffering therapy could enhance the ICI response by restoring immune cell activity, whereas CAIX inhibitors could be paired with chemotherapy or radiation to improve treatment outcomes. Additionally, integrating pH modulation with lactate transport inhibitors (e.g., MCT inhibitors) could simultaneously target both TME acidity and metabolic dependencies, offering a comprehensive approach to disrupting tumor-driven immune evasion (103, 120).

Addressing these limitations and enhancing the understanding of the molecular mechanisms involved in pH regulation are essential for promoting the efficacy of TME-targeted therapies and improving cancer treatment outcomes. This includes examining the impact of pH variations on molecular pathways, including immune cell activation, cytokine production, and immune checkpoint regulation. The role of pH regulation in tumor angiogenesis, extracellular matrix remodeling, and drug resistance in treatment modalities, including chemotherapy and immunotherapy, requires further investigation. Investigating these unresolved molecular interactions is crucial for advancing pH-targeted therapies and improving their clinical efficacy.

#### 2.2.2 Targeted lactate transport intervention: therapeutic potential of MCT inhibitors

Lactate transport plays a pivotal role in tumor metabolic reprogramming, primarily mediated by MCT1 and MCT4. These transporters help sustain glycolysis, regenerate NAD^+^, and maintain an acidic TME, all of which promote tumor growth and immune suppression. Given their crucial role, MCTs represent promising therapeutic targets for altering tumor metabolism and alleviating immune suppression. The MCT inhibitors are listed in **Table 3**.

**Table 3:** LDH and MCT inhibitors.

Mechanism of action	Compound	Cancers	Research status
MCT1/4	AZD3965	Breast and colon cancer, lymphoma xenografts, human diffuse large B-cell lymphomas, human B-cell lymphoma, lymphoblast, B-cell non-Hodgkin lymphoma, Raji Burkitt’s lymphoma cells	Phase I and Preclinical (121, 122)
	α-Cyano-4-hydroxycinnamate	Glioma, breast cancer, pancreatic ductal adenocarcinoma	Preclinical (176–178)
	Syrosingopine	Breast, colon, cervical cancer, and leukemia cells	Preclinical (124)
	BAY-8002	Diffuse large B cell lymphoma	Preclinical (179)
	7ACC2	Pancreatic adenocarcinoma	Preclinical (180)
	AR-C155858	Breast cancer tumor xenografts	Preclinical (181)
	Diclofenac	Melanoma, cervical cancer, bladder cancer, colorectal cancer	Preclinical (126, 182)
	Gossypol (NCT02697344)	Multiple myeloma	Phase I
LDHA/LDHB	Galloflavin	Colorectal, endometrial, and breast cancer	Preclinical (143–145)
	1-(Phenylseleno)- 4-(Trifluoromethyl) Benzene	Large cell lung cancer, breast cancer, hepatocellular carcinoma, malignant melanoma, colorectal adenocarcinoma, murine lung cancer cells	Preclinical (183)
	Oxamate	Colorectal, pancreatic, gastric, and non-small cell lung cancer	Preclinical (136–139)
	GSK 2837808A	Nasopharyngeal carcinoma, pancreatic cancer xenografts	Preclinical (184, 185)
	AZ-33	Breast cancer	Preclinical (186)
	GNE-140	Breast, colorectal adenocarcinoma, and lung cancer, glioma xenografts	Preclinical (187–189)
	Compound 24	Pancreas carcinoma	Preclinical (190)
	Compounds 5 and 11	Osteosarcoma	Preclinical (191)

LDH, lactate dehydrogenase; MCT, monocarboxylate transporter.

MCT1 inhibition has shown significant preclinical potential. AZD3965, a specific MCT1 inhibitor, increases intracellular lactate levels, disrupts glycolysis, and induces tumor cell death (121, 122). In lymphoma and small-cell lung cancer models, AZD3965 substantially reduced tumor growth and is currently being evaluated in a phase I clinical study (NCT01791595) (123). However, the compensatory effect of MCT4 can reduce the effectiveness of MCT1-targeted therapies. To address this issue, dual MCT1/MCT4 inhibition strategies were explored. For instance, syrosingopine inhibits lactate transport through multiple pathways, and when combined with metabolic modulators such as metformin, it enhances antitumor activity in solid tumor models (124).

In addition to direct tumor cytotoxicity, MCT inhibitors have significant immunomodulatory effects. MCT1 promotes lactate uptake in Tregs and TAMs, thereby maintaining their immunosuppressive phenotype. Inhibiting MCT1 reduces Treg accumulation in lactate-rich TMEs, enhancing CD8^+^ T cell activity and restoring antitumor immunity. In preclinical models, MCT4 inhibition was shown to enhance NK cell function, thereby improving tumor control (139, 140). Similarly, it can reduce the acidification of the TME, upregulate chemokines such as CXCL9 and CXCL10, and improve the responses to ICIs in hepatocellular cancer models (125). Recently, diclofenac was demonstrated to inhibit MCT1 and MCT4, thereby reducing glycolytic activity and lactate accumulation within the tumor microenvironment. This effect showed that diclofenac not only reverses tumor acidosis but also preserves T cell effector functions, enhancing the efficacy of ICIs such as anti-PD1 treatment (126).

Selective MCT1 inhibition (e.g., AZD3965) effectively disrupts lactate efflux and tumor metabolism but can be counteracted by MCT4 compensation, limiting its standalone efficacy. Preclinical studies have shown that the combination of AZD3965 and ICIs can produce encouraging results (127). Dual inhibition (e.g., syrosingopine or diclofenac) overcomes this limitation but increases the risk of systemic toxicity, as MCTs are essential for lactate transport in normal cells, such as erythrocytes and muscle cells (123, 128). Combination strategies may enhance treatment efficacy while mitigating these drawbacks. For example, MCT inhibitors could be paired with ICIs to restore immune cell function by lowering lactate-driven immunosuppression, improving antitumor immunity. Additionally, combining MCT inhibition with metabolic modulators, such as metformin, may amplify metabolic stress in tumor cells, leading to more pronounced therapeutic effects (129).

Despite these advancements, resistance and toxicity remain significant barriers to clinical application. Resistance often arises from compensatory metabolic changes, whereas systemic toxicity affects lactate-dependent normal tissues. Future research should focus on enhancing the selectivity of MCT inhibitors, exploring combination therapies with ICIs or glycolysis modulators, and employing nanoparticle delivery systems to improve tumor-specific targeting (130, 131). By addressing these challenges, MCT-targeted therapies can substantially improve cancer treatment outcomes by modifying tumor metabolism and restoring immune function. Furthermore, combining lactate-targeted strategies with ICIs offers a promising approach to counteract TME-induced immunosuppression, as discussed below.

#### 2.2.3 Targeting LDH: Dual potential in metabolic and immune regulation

LDHA plays a crucial role in tumor metabolism by converting pyruvate to lactate, replenishing NAD^+^ for glycolysis, and acidifying the TME, all of which promote immune evasion and tumor growth. These functions render LDHA an appealing therapeutic target for disrupting tumor metabolic pathways and reducing TME-driven immunosuppression (132–135). LDH-targeting drugs are shown in **Table 2**.

Studies targeting LDHA have shown promising results in preclinical models. For example, both Oxamate (a competitive inhibitor) and FX11 (an inhibitor targeting the NADH binding site) reduced lactate production, decreased TME acidity, and inhibited tumor growth in gastric, lymphoma, and pancreatic cancers (136–142). In addition, the combination of FX11 with the NAD^+^ production inhibitor FK866 enhanced antitumor activity via a dual metabolic blockade. New inhibitors, such as Galloflavin and N-hydroxyindole (NHI) derivatives, further expand LDHA inhibition strategies. Galloflavin has shown significant lactic acid inhibition in preclinical trials, whereas NHI derivatives address resistance to single-target inhibitors by targeting both LDHA and LDHB (143–146). Combining LDHA inhibitors with other therapies, such as metformin, has also shown promise in significantly inhibiting melanoma growth by increasing metabolic stress in tumor cells. Targeting LDHB offers additional therapeutic opportunities for cancers that rely on autophagy and lysosomal pathways (147, 148).

Despite these promising findings, a more critical evaluation of LDHA-targeted therapies is needed, particularly regarding the high drug concentrations required for effective inhibition because of the strong expression of LDHA in tumors (149). These high concentrations may lead to off-target effects in glycolysis-dependent healthy tissues, such as CTLs and NK cells, which could limit the overall efficacy of tumor immune control. Moreover, the strong expression of LDHA in immune cells like CTLs and NK cells can impede their effector functions, thus reducing the therapeutic benefit of LDHA inhibition in immunotherapy. Future research should focus on developing selective inhibitors that can specifically target tumor cells while minimizing toxicity to immune cells, as well as exploring combination strategies with ICIs to enhance the therapeutic impact of LDHA inhibitors (150). Furthermore, advanced delivery technologies, such as nanoparticles, could help improve drug specificity and reduce systemic side effects (130).

#### 2.2.4 Lithium carbonate-mediated metabolic reprogramming to enhance immune function

As mentioned previously, lactate can function as a carbon source to enhance oxidative phosphorylation in T cells. The role of lithium carbonate (LC) in this metabolic reprogramming process has been shown to significantly improve T-cell function in the TME. LC enhances T-cell function in the tumor microenvironment by facilitating lactate entry into mitochondria for ATP production, shifting energy metabolism from glycolysis to oxidative phosphorylation. This process improves mitochondrial function, counteracts lactate-induced immunosuppression, and supports T-cell survival and anti-tumor activity. LC also facilitates long-term T-cell memory formation and persistence in tumors, thereby addressing a major limitation of current cancer immunotherapies. In preclinical models, LC treatment was found to restore the effector functions of CD8^+^ T cells, increasing their ability to kill tumor cells and improving tumor control (151).

The ability to switch between different metabolic pathways based on carbon source availability is regulated by key signaling pathways such as mTORC1 and AMPK (152). These pathways control the balance between glycolysis and oxidative phosphorylation, directly affecting T-cell differentiation and function. Lithium carbonate’s ability to enhance oxidative metabolism and reduce lactate-mediated suppression of T-cell function makes it a promising tool for enhancing the efficacy of ICIs and other immunotherapies (153).

In summary, promoting the mitochondrial use of lactate, as observed with lithium carbonate treatment, is a powerful strategy to boost CD8^+^ T cell metabolism, function, and persistence within the TME. This approach provides a comprehensive method to reprogram T cells, enhance their anti-tumor activity, and potentially overcome the metabolic barriers that hinder current immunotherapeutic strategies. By targeting the metabolic pathways that regulate T-cell function, lithium carbonate offers a novel therapeutic route for improving T-cell-based therapies in cancer treatment. Nonetheless, as noted earlier, regulatory T cells, a specific subset of T cells, can be activated in environments with elevated lactate levels. In the therapeutic approaches outlined in this section, these cells may also be stimulated. Their immunosuppressive and tumor-enhancing properties could adversely affect treatment efficacy or result in other unforeseen complex outcomes. Consequently, these therapeutic strategies warrant additional research.

In conclusion, although current pharmacological strategies targeting lactate show good potential, they also face significant challenges, largely because the effects of these drugs tend to be widespread and not limited to specific cell populations. Therefore, to provide a clearer understanding of their therapeutic perspectives, we summarized the main strengths and limitations of the four lactate-targeted therapeutics mentioned above in **Table 4**.

**Table 4:** Advantages and disadvantages of lactate-targeted therapies.

Therapies	Advantages	Disadvantages
Neutralizing Acidity	Enhancement of immune cell function, improvement of ICI efficacy, and potential for multimodal therapy	Non-specific effects, potential toxicity, limited efficacy
MCT Inhibition	Direct targeting of tumor metabolism, immunomodulatory effects, and enhanced ICI efficacy	Compensation mechanisms, systemic toxicity, risk of drug resistance
Targeting LDH	Dual mechanism of action, combined therapeutic potential, multi-target inhibition	High concentration requirement, immune cell suppression, risk of drug resistance
Lithium Carbonate-Mediated Metabolic Reprogramming	Synergistic effect, enhances ICI efficacy, possesses long-term immune memory, well-supported by extensive preclinical validation	Risk of resistance, clinical translation challenges
Integration of Lactate-Related Pathways with Immunotherapy	Synergistic effect, improves T cell function	Risk of resistance, clinical translation challenges

## 3 Conclusions

Lactate metabolism plays a crucial role in tumor development and immune evasion and acts as a link between tumor metabolic reprogramming and the immunosuppressive TME. Targeting lactate metabolism presents a promising therapeutic approach, with preclinical data showing its potential to alter tumor glycolysis, reduce TME acidity, and boost antitumor immune responses. Strategies such as LDH and MCT inhibitors, particularly when combined with ICIs, have demonstrated synergistic benefits, paving the way for clinical application.

Future research should focus on improving the specificity of lactate-targeting therapies, exploring their role in various cancer types, and combining them with emerging treatments, such as CAR-T therapy. Developing biomarkers to guide patient selection is crucial for optimizing treatment outcomes. A deeper understanding of lactate metabolism and its integration with immunotherapy has the potential to transform cancer treatment by advancing precision medicine.

## References

[1] Zhang W Wang C Hu X Lian Y Ding C Ming L. Inhibition of LDHA suppresses cell proliferation and increases mitochondrial apoptosis via the JNK signaling pathway in cervical cancer cells. Oncol Rep. (2022) 47(4):77. doi: 10.3892/or.2022.8288 35191522 PMC8892607

[2] Wen L Han Z Li J Du Y. c-MYC and HIF1α promoter G-quadruplexes dependent metabolic regulation mechanism of berberine in colon cancer. J Gastrointest Oncol. (2022) 13:1152–68. doi: 10.21037/jgo-22-389 PMC9274050 35837174

[3] Duan Q Zhang S Wang Y Lu D Sun Y Wu Y. Proton-coupled monocarboxylate transporters in cancer: From metabolic crosstalk, immunosuppression and anti-apoptosis to clinical applications. Front Cell Dev Biol. (2022) 10:1069555. doi: 10.3389/fcell.2022.1069555 36506099 PMC9727313

[4] Boidot R Végran F Meulle A Le Breton A Dessy C Sonveaux P. Regulation of monocarboxylate transporter MCT1 expression by p53 mediates inward and outward lactate fluxes in tumors. Cancer Res. (2012) 72:939–48. doi: 10.1158/0008-5472.CAN-11-2474 22184616

[5] Rahman MA Yadab MK Ali MM. Emerging role of extracellular pH in tumor microenvironment as a therapeutic target for cancer immunotherapy. Cells. (2024) 13(22):1924. doi: 10.3390/cells13221924 39594672 PMC11592846

[6] Zhou Y Lou J Tian Y Ding J Wang X Tang B. How lactate affects immune strategies in lymphoma. Front Mol Biosci. (2024) 11:1480884. doi: 10.3389/fmolb.2024.1480884 39464313 PMC11502318

[7] Zhang Y Zhai Z Duan J Wang X Zhong J Wu L. Lactate: the mediator of metabolism and immunosuppression. Front Endocrinol (Lausanne). (2022) 13:901495. doi: 10.3389/fendo.2022.901495 35757394 PMC9218951

[8] Zhang D Tang Z Huang H Zhou G Cui C Weng Y. Metabolic regulation of gene expression by histone lactylation. Nature. (2019) 574:575–80. doi: 10.1038/s41586-019-1678-1 PMC6818755 31645732

[9] Lu Y Zhu J Zhang Y Li W Xiong Y Fan Y. Lactylation-driven IGF2BP3-mediated serine metabolism reprogramming and RNA m6A-modification promotes lenvatinib resistance in HCC. Adv Sci (Weinh). (2024) 11:e2401399. doi: 10.1002/advs.202401399 39450426 PMC11633555

[10] Xie Y Hu H Liu M Zhou T Cheng X Huang W. The role and mechanism of histone lactylation in health and diseases. Front Genet. (2022) 13:949252. doi: 10.3389/fgene.2022.949252 36081996 PMC9445422

[11] Hu Y He Z Li Z Wang Y Wu N Sun H. Lactylation: the novel histone modification influence on gene expression, protein function, and disease. Clin Epigenetics. (2024) 16:72. doi: 10.1186/s13148-024-01682-2 38812044 PMC11138093

[12] Qu J Li P Sun Z. Histone lactylation regulates cancer progression by reshaping the tumor microenvironment. Front Immunol. (2023) 14:1284344. doi: 10.3389/fimmu.2023.1284344 37965331 PMC10641494

[13] Walenta S Wetterling M Lehrke M Schwickert G Sundfør K Rofstad EK. High lactate levels predict likelihood of metastases, tumor recurrence, and restricted patient survival in human cervical cancers. Cancer Res. (2000) 60:916–21. 10706105

[14] de-la-Cruz-López KG Castro-Muñoz LJ Reyes-Hernández DO García-Carrancá A Manzo-Merino J. Lactate in the regulation of tumor microenvironment and therapeutic approaches. Front Oncol. (2019) 9:1143. doi: 10.3389/fonc.2019.01143 31737570 PMC6839026

[15] Marchiq I Pouysségur J. Hypoxia, cancer metabolism and the therapeutic benefit of targeting lactate/H(+) symporters. J Mol Med (Berl). (2016) 94:155–71. doi: 10.1007/s00109-015-1307-x PMC4762928 26099350

[16] Walenta S Salameh A Lyng H Evensen JF Mitze M Rofstad EK. Correlation of high lactate levels in head and neck tumors with incidence of metastasis. Am J Pathol. (1997) 150:409–15. PMC1858285 9033256

[17] Kennedy KM Scarbrough PM Ribeiro A Richardson R Yuan H Sonveaux P. Catabolism of exogenous lactate reveals it as a legitimate metabolic substrate in breast cancer. PloS One. (2013) 8:e75154. doi: 10.1371/journal.pone.0075154 24069390 PMC3771963

[18] Jin R Neufeld L McGaha TL. Linking macrophage metabolism to function in the tumor microenvironment. Nat Cancer. (2025) 6:239–52. doi: 10.1038/s43018-025-00909-2 39962208

[19] Quinn WJ 3rd Jiao J TeSlaa T Stadanlick J Wang Z Wang L. Lactate limits T cell proliferation via the NAD(H) redox state. Cell Rep. (2020) 33:108500. doi: 10.1016/j.celrep.2020.108500 33326785 PMC7830708

[20] Lopez E Karattil R Nannini F Weng-Kit-Cheung G Denzler L Galvez-Cancino F. Inhibition of lactate transport by MCT-1 blockade improves chimeric antigen receptor T-cell therapy against B-cell Malignancies. J Immunother Cancer. (2023) 11(6):e006287. doi: 10.1136/jitc-2022-006287 37399358 PMC10314680

[21] Brand A Singer K Koehl GE Kolitzus M Schoenhammer G Thiel A. LDHA-associated lactic acid production blunts tumor immunosurveillance by T and NK cells. Cell Metab. (2016) 24:657–71. doi: 10.1016/j.cmet.2016.08.011 27641098

[22] Ganjoo S Gupta P Corbali HI Nanez S Riad TS Duong LK. The role of tumor metabolism in modulating T-Cell activity and in optimizing immunotherapy. Front Immunol. (2023) 14:1172931. doi: 10.3389/fimmu.2023.1172931 37180129 PMC10169689

[23] Buck MD O’Sullivan D Klein Geltink RI Curtis JD Chang CH Sanin DE. Mitochondrial dynamics controls T cell fate through metabolic programming. Cell. (2016) 166:63–76. doi: 10.1016/j.cell.2016.05.035 27293185 PMC4974356

[24] Uhl FM Chen S O’Sullivan D Edwards-Hicks J Richter G Haring E. Metabolic reprogramming of donor T cells enhances graft-versus-leukemia effects in mice and humans. Sci Transl Med. (2020) 12(567):eabb8969. doi: 10.1126/scitranslmed.abb8969 33115954 PMC8529950

[25] Faubert B Li KY Cai L Hensley CT Kim J Zacharias LG. Lactate metabolism in human lung tumors. Cell. (2017) 171:358–71.e9. doi: 10.1016/j.cell.2017.09.019 28985563 PMC5684706

[26] Hui S Ghergurovich JM Morscher RJ Jang C Teng X Lu W. Glucose feeds the TCA cycle via circulating lactate. Nature. (2017) 551:115–8. doi: 10.1038/nature24057 PMC5898814 29045397

[27] Tasdogan A Faubert B Ramesh V Ubellacker JM Shen B Solmonson A. Metabolic heterogeneity confers differences in melanoma metastatic potential. Nature. (2020) 577:115–20. doi: 10.1038/s41586-019-1847-2 PMC6930341 31853067

[28] Kaymak I Luda KM Duimstra LR Ma EH Longo J Dahabieh MS. Carbon source availability drives nutrient utilization in CD8(+) T cells. Cell Metab. (2022) 34:1298–311.e6. doi: 10.1016/j.cmet.2022.07.012 35981545 PMC10068808

[29] Mehta MM Weinberg SE Chandel NS. Mitochondrial control of immunity: beyond ATP. Nat Rev Immunol. (2017) 17:608–20. doi: 10.1038/nri.2017.66 28669986

[30] O’Neill LA Kishton RJ Rathmell J. A guide to immunometabolism for immunologists. Nat Rev Immunol. (2016) 16:553–65. doi: 10.1038/nri.2016.70 PMC5001910 27396447

[31] San-Millán I Julian CG Matarazzo C Martinez J Brooks GA. Is lactate an oncometabolite? Evidence supporting a role for lactate in the regulation of transcriptional activity of cancer-related genes in MCF7 breast cancer cells. Front Oncol. (2019) 9:1536. doi: 10.3389/fonc.2019.01536 32010625 PMC6971189

[32] Feng J Yang H Zhang Y Wei H Zhu Z Zhu B. Tumor cell-derived lactate induces TAZ-dependent upregulation of PD-L1 through GPR81 in human lung cancer cells. Oncogene. (2017) 36:5829–39. doi: 10.1038/onc.2017.188 28604752

[33] Comito G Iscaro A Bacci M Morandi A Ippolito L Parri M. Lactate modulates CD4(+) T-cell polarization and induces an immunosuppressive environment, which sustains prostate carcinoma progression via TLR8/miR21 axis. Oncogene. (2019) 38:3681–95. doi: 10.1038/s41388-019-0688-7 30664688

[34] Angelin A Gil-de-Gómez L Dahiya S Jiao J Guo L Levine MH. Foxp3 reprograms T cell metabolism to function in low-glucose, high-lactate environments. Cell Metab. (2017) 25:1282–93.e7. doi: 10.1016/j.cmet.2016.12.018 28416194 PMC5462872

[35] Haas R Smith J Rocher-Ros V Nadkarni S Montero-Melendez T D’Acquisto F. Lactate regulates metabolic and pro-inflammatory circuits in control of T cell migration and effector functions. PloS Biol. (2015) 13:e1002202. doi: 10.1371/journal.pbio.1002202 26181372 PMC4504715

[36] Wang Z Dai Z Zhang H Liang X Zhang X Wen Z. Tumor-secreted lactate contributes to an immunosuppressive microenvironment and affects CD8 T-cell infiltration in glioblastoma. Front Immunol. (2023) 14:894853. doi: 10.3389/fimmu.2023.894853 37122693 PMC10130393

[37] Kumar A Pyaram K Yarosz EL Hong H Lyssiotis CA Giri S. Enhanced oxidative phosphorylation in NKT cells is essential for their survival and function. Proc Natl Acad Sci U S A. (2019) 116:7439–48. doi: 10.1073/pnas.1901376116 PMC6462103 30910955

[38] Harmon C Robinson MW Hand F Almuaili D Mentor K Houlihan DD. Lactate-mediated acidification of tumor microenvironment induces apoptosis of liver-resident NK cells in colorectal liver metastasis. Cancer Immunol Res. (2019) 7:335–46. doi: 10.1158/2326-6066.CIR-18-0481 30563827

[39] Xie D Zhu S Bai L. Lactic acid in tumor microenvironments causes dysfunction of NKT cells by interfering with mTOR signaling. Sci China Life Sci. (2016) 59:1290–6. doi: 10.1007/s11427-016-0348-7 27995420

[40] Claps G Faouzi S Quidville V Chehade F Shen S Vagner S. The multiple roles of LDH in cancer. Nat Rev Clin Oncol. (2022) 19:749–62. doi: 10.1038/s41571-022-00686-2 36207413

[41] Hu Z Xu X Wei H. The adverse impact of tumor microenvironment on NK-cell. Front Immunol. (2021) 12:633361. doi: 10.3389/fimmu.2021.633361 34177887 PMC8226132

[42] Wang L Chen Z Liu G Pan Y. Functional crosstalk and regulation of natural killer cells in tumor microenvironment: Significance and potential therapeutic strategies. Genes Dis. (2023) 10:990–1004. doi: 10.1016/j.gendis.2022.07.009 37396514 PMC10308134

[43] Gottfried E Kunz-Schughart LA Ebner S Mueller-Klieser W Hoves S Andreesen R. Tumor-derived lactic acid modulates dendritic cell activation and antigen expression. Blood. (2006) 107:2013–21. doi: 10.1182/blood-2005-05-1795 16278308

[44] Caronni N Simoncello F Stafetta F Guarnaccia C Ruiz-Moreno JS Opitz B. Downregulation of membrane trafficking proteins and lactate conditioning determine loss of dendritic cell function in lung cancer. Cancer Res. (2018) 78:1685–99. doi: 10.1158/0008-5472.CAN-17-1307 29363545

[45] Yao Y Li W Kaplan MH Chang CH. Interleukin (IL)-4 inhibits IL-10 to promote IL-12 production by dendritic cells. J Exp Med. (2005) 201:1899–903. doi: 10.1084/jem.20050324 PMC2212025 15967820

[46] Manoharan I Prasad PD Thangaraju M Manicassamy S. Lactate-dependent regulation of immune responses by dendritic cells and macrophages. Front Immunol. (2021) 12:e26. doi: 10.3389/fimmu.2021.691134 PMC8358770 34394085

[47] Feichtinger RG Lang R. Targeting L-lactate metabolism to overcome resistance to immune therapy of melanoma and other tumor entities. J Oncol. (2019) 2019:2084195. doi: 10.1155/2019/2084195 31781212 PMC6875281

[48] Ishihara S Hata K Hirose K Okui T Toyosawa S Uzawa N. The lactate sensor GPR81 regulates glycolysis and tumor growth of breast cancer. Sci Rep. (2022) 12:6261. doi: 10.1038/s41598-022-10143-w 35428832 PMC9012857

[49] Ranganathan P Shanmugam A Swafford D Suryawanshi A Bhattacharjee P Hussein MS. GPR81, a cell-surface receptor for lactate, regulates intestinal homeostasis and protects mice from experimental colitis. J Immunol. (2018) 200:1781–9. doi: 10.4049/jimmunol.1700604 PMC5858928 29386257

[50] Guak H Al Habyan S Ma EH Aldossary H Al-Masri M Won SY. Glycolytic metabolism is essential for CCR7 oligomerization and dendritic cell migration. Nat Commun. (2018) 9:2463. doi: 10.1038/s41467-018-04804-6 29941886 PMC6018630

[51] Plebanek MP Xue Y Nguyen YV DeVito NC Wang X Holtzhausen A. A lactate-SREBP2 signaling axis drives tolerogenic dendritic cell maturation and promotes cancer progression. Sci Immunol. (2024) 9:eadi4191. doi: 10.1126/sciimmunol.adi4191 38728412 PMC11926670

[52] Multhoff G Vaupel P. Lactate-avid regulatory T cells: metabolic plasticity controls immunosuppression in tumour microenvironment. Signal Transduction Targeted Ther. (2021) 6:171. doi: 10.1038/s41392-021-00598-0 PMC8087677 33931598

[53] Zhou J Shao Q Lu Y Li Y Xu Z Zhou B. Monocarboxylate transporter upregulation in induced regulatory T cells promotes resistance to anti-PD-1 therapy in hepatocellular carcinoma patients. Front Oncol. (2022) 12:960066. doi: 10.3389/fonc.2022.960066 35965549 PMC9368998

[54] Watson MJ Vignali PDA Mullett SJ Overacre-Delgoffe AE Peralta RM Grebinoski S. Metabolic support of tumour-infiltrating regulatory T cells by lactic acid. Nature. (2021) 591:645–51. doi: 10.1038/s41586-020-03045-2 PMC7990682 33589820

[55] Su J Mao X Wang L Chen Z Wang W Zhao C. Lactate/GPR81 recruits regulatory T cells by modulating CX3CL1 to promote immune resistance in a highly glycolytic gastric cancer. Oncoimmunology. (2024) 13:2320951. doi: 10.1080/2162402X.2024.2320951 38419759 PMC10900271

[56] Gan X Zhang R Gu J Ju Z Wu X Wang Q. Acidic microenvironment regulates the severity of hepatic ischemia/reperfusion injury by modulating the generation and function of tregs via the PI3K-mTOR pathway. Front Immunol. (2019) 10:2945. doi: 10.3389/fimmu.2019.02945 31998287 PMC6962105

[57] Liu Z Zhang Z Zhang Y Zhou W Zhang X Peng C. Spatial transcriptomics reveals that metabolic characteristics define the tumor immunosuppression microenvironment via iCAF transformation in oral squamous cell carcinoma. Int J Oral Sci. (2024) 16:9. doi: 10.1038/s41368-023-00267-8 38287007 PMC10824761

[58] Fu S He K Tian C Sun H Zhu C Bai S. Impaired lipid biosynthesis hinders anti-tumor efficacy of intratumoral iNKT cells. Nat Commun. (2020) 11:438. doi: 10.1038/s41467-020-14332-x 31974378 PMC6978340

[59] Rao D Stunnenberg JA Lacroix R Dimitriadis P Kaplon J Verburg F. Acidity-mediated induction of FoxP3(+) regulatory T cells. Eur J Immunol. (2023) 53:e2250258. doi: 10.1002/eji.202250258 36788428

[60] Yang X Lu Y Hang J Zhang J Zhang T Huo Y. Lactate-modulated immunosuppression of myeloid-derived suppressor cells contributes to the radioresistance of pancreatic cancer. Cancer Immunol Res. (2020) 8:1440–51. doi: 10.1158/2326-6066.CIR-20-0111 32917658

[61] Greten TF Manns MP Korangy F. Myeloid derived suppressor cells in human diseases. Int immunopharmacology. (2011) 11:802–7. doi: 10.1016/j.intimp.2011.01.003 PMC3478130 21237299

[62] Vitale M Cantoni C Pietra G Mingari MC Moretta L. Effect of tumor cells and tumor microenvironment on NK-cell function. Eur J Immunol. (2014) 44:1582–92. doi: 10.1002/eji.201344272 24777896

[63] Morrot A da Fonseca LM Salustiano EJ Gentile LB Conde L Filardy AA. Metabolic symbiosis and immunomodulation: how tumor cell-derived lactate may disturb innate and adaptive immune responses. Front Oncol. (2018) 8:81. doi: 10.3389/fonc.2018.00081 29629338 PMC5876249

[64] Veglia F Sanseviero E Gabrilovich DI. Myeloid-derived suppressor cells in the era of increasing myeloid cell diversity. Nat Rev Immunol. (2021) 21:485–98. doi: 10.1038/s41577-020-00490-y PMC7849958 33526920

[65] Kim J Choi JY Min H Hwang KW. Exploring the potential of glycolytic modulation in myeloid-derived suppressor cells for immunotherapy and disease management. Immune Netw. (2024) 24(3):7575. doi: 10.4110/in.2024.24.e26 PMC11224668 38974210

[66] Zhi S Chen C Huang H Zhang Z Zeng F Zhang S. Hypoxia-inducible factor in breast cancer: role and target for breast cancer treatment. Front Immunol. (2024) 15:1370800. doi: 10.3389/fimmu.2024.1370800 38799423 PMC11116789

[67] Zhao JL Ye YC Gao CC Wang L Ren KX Jiang R. Notch-mediated lactate metabolism regulates MDSC development through the Hes1/MCT2/c-Jun axis. Cell Rep. (2022) 38:110451. doi: 10.1016/j.celrep.2022.110451 35263597

[68] Li W Tanikawa T Kryczek I Xia H Li G Wu K. Aerobic glycolysis controls myeloid-derived suppressor cells and tumor immunity via a specific CEBPB isoform in triple-negative breast cancer. Cell Metab. (2018) 28:87–103.e6. doi: 10.1016/j.cmet.2018.04.022 29805099 PMC6238219

[69] Khatib-Massalha E Bhattacharya S Massalha H Biram A Golan K Kollet O. Lactate released by inflammatory bone marrow neutrophils induces their mobilization via endothelial GPR81 signaling. Nat Commun. (2020) 11:3547. doi: 10.1038/s41467-020-17402-2 32669546 PMC7363928

[70] Jedlička M Feglarová T Janstová L Hortová-Kohoutková M Frič J. Lactate from the tumor microenvironment - A key obstacle in NK cell-based immunotherapies. Front Immunol. (2022) 13:932055. doi: 10.3389/fimmu.2022.932055 36330529 PMC9623302

[71] Han S Bao X Zou Y Wang L Li Y Yang L. amp]]lt;span class=“smallcaps smallerCapital”>d-lactate modulates M2 tumor-associated macrophages and remodels immunosuppressive tumor microenvironment for hepatocellular carcinoma. Sci Adv. (2023) 9(29):eadg2697. doi: 10.1126/sciadv.adg2697 37467325 PMC10355835

[72] Jiang R Ren W-J Wang L-Y Zhang W Jiang Z-H Zhu G-Y. Targeting lactate: an emerging strategy for macrophage regulation in chronic inflammation and cancer. Biomolecules. (2024) 14:1202. doi: 10.3390/biom14101202 39456135 PMC11505598

[73] Xu B Liu Y Li N Geng Q. Lactate and lactylation in macrophage metabolic reprogramming: current progress and outstanding issues. Front Immunol. (2024) 15:1395786. doi: 10.3389/fimmu.2024.1395786 38835758 PMC11148263

[74] Zhou HC Xin-Yan Y Yu WW Liang XQ Du XY Liu ZC. Lactic acid in macrophage polarization: The significant role in inflammation and cancer. Int Rev Immunol. (2022) 41:4–18. doi: 10.1080/08830185.2021.1955876 34304685

[75] Gao J Liang Y Wang L. Shaping polarization of tumor-associated macrophages in cancer immunotherapy. Front Immunol. (2022) 13:888713. doi: 10.3389/fimmu.2022.888713 35844605 PMC9280632

[76] Hasan MN Capuk O Patel SM Sun D. The role of metabolic plasticity of tumor-associated macrophages in shaping the tumor microenvironment immunity. Cancers. (2022) 14:3331. doi: 10.3390/cancers14143331 35884391 PMC9316955

[77] Chen S Saeed AFUH Liu Q Jiang Q Xu H Xiao GG. Macrophages in immunoregulation and therapeutics. Signal Transduction Targeted Ther. (2023) 8:207. doi: 10.1038/s41392-023-01452-1 PMC10200802 37211559

[78] Zheng J. Energy metabolism of cancer: Glycolysis versus oxidative phosphorylation (Review). Oncol Lett. (2012) 4:1151–7. doi: 10.3892/ol.2012.928 PMC3506713 23226794

[79] Li X Yang Y Zhang B Lin X Fu X An Y. Lactate metabolism in human health and disease. Signal Transduction Targeted Ther. (2022) 7:305. doi: 10.1038/s41392-022-01151-3 PMC9434547 36050306

[80] Van den Bossche J Baardman J Otto NA van der Velden S Neele AE van den Berg SM. Mitochondrial dysfunction prevents repolarization of inflammatory macrophages. Cell Rep. (2016) 17:684–96. doi: 10.1016/j.celrep.2016.09.008 27732846

[81] Van den Bossche J Baardman J de Winther MP. Metabolic characterization of polarized M1 and M2 bone marrow-derived macrophages using real-time extracellular flux analysis. J visualized experiments: JoVE. (2015) 105:53424. doi: 10.3791/53424 PMC4692751 26649578

[82] Noe JT Rendon BE Geller AE Conroy LR Morrissey SM Young LEA. Lactate supports a metabolic-epigenetic link in macrophage polarization. Sci Adv. (2021) 7:eabi8602. doi: 10.1126/sciadv.abi8602 34767443 PMC8589316

[83] Herzig S Raemy E Montessuit S Veuthey JL Zamboni N Westermann B. Identification and functional expression of the mitochondrial pyruvate carrier. Science. (2012) 337:93–6. doi: 10.1126/science.1218530 22628554

[84] Pan Y Yu Y Wang X Zhang T. Tumor-associated macrophages in tumor immunity. Front Immunol. (2020) 11:583084. doi: 10.3389/fimmu.2020.583084 33365025 PMC7751482

[85] Sun K Shen Y Xiao X Xu H Zhang Q Li M. Crosstalk between lactate and tumor-associated immune cells: clinical relevance and insight. Front Oncol. (2024) 14:1506849. doi: 10.3389/fonc.2024.1506849 39678492 PMC11638036

[86] Raychaudhuri D Singh P Hennessey M Tannir AJ Chakraborty B Natarajan SM. Histone lactylation drives CD8 T cell metabolism and function. Nat Immunol. (2024) 25(11):2140–51. doi: 10.1038/s41590-024-01985-9 PMC13211864 39375549

[87] Elion DL Cook RS. Harnessing RIG-I and intrinsic immunity in the tumor microenvironment for therapeutic cancer treatment. Oncotarget. (2018) 9:29007–17. doi: 10.18632/oncotarget.25626 PMC6034747 29989043

[88] Linares JF Cid-Diaz T Duran A Osrodek M Martinez-Ordoñez A Reina-Campos M. The lactate-NAD(+) axis activates cancer-associated fibroblasts by downregulating p62. Cell Rep. (2022) 39:110792. doi: 10.1016/j.celrep.2022.110792 35545049 PMC9136538

[89] Gu X Zhu Y Su J Wang S Su X Ding X. Lactate-induced activation of tumor-associated fibroblasts and IL-8-mediated macrophage recruitment promote lung cancer progression. Redox Biol. (2024) 74:103209. doi: 10.1016/j.redox.2024.103209 38861833 PMC11215341

[90] Chung JY Chan MK Li JS Chan AS Tang PC Leung KT. TGF-β Signaling: from tissue fibrosis to tumor microenvironment. Int J Mol Sci. (2021) 22(14):7575. doi: 10.3390/ijms22147575 34299192 PMC8303588

[91] Ohnuki H Jiang K Wang D Salvucci O Kwak H Sánchez-Martín D. Tumor-infiltrating myeloid cells activate Dll4/Notch/TGF-β signaling to drive Malignant progression. Cancer Res. (2014) 74:2038–49. doi: 10.1158/0008-5472.CAN-13-3118 PMC3985086 24520074

[92] Chen AN Luo Y Yang YH Fu JT Geng XM Shi JP. Lactylation, a novel metabolic reprogramming code: current status and prospects. Front Immunol. (2021) 12:688910. doi: 10.3389/fimmu.2021.688910 34177945 PMC8222712

[93] Wei S Zhang J Zhao R Shi R An L Yu Z. Histone lactylation promotes Malignant progression by facilitating USP39 expression to target PI3K/AKT/HIF-1α signal pathway in endometrial carcinoma. Cell Death Discovery. (2024) 10:121. doi: 10.1038/s41420-024-01898-4 38459014 PMC10923933

[94] Zhang Y Song H Li M Lu P. Histone lactylation bridges metabolic reprogramming and epigenetic rewiring in driving carcinogenesis: Oncometabolite fuels oncogenic transcription. Clin Transl Med. (2024) 14:e1614. doi: 10.1002/ctm2.v14.3 38456209 PMC10921234

[95] Huang ZW Zhang XN Zhang L Liu LL Zhang JW Sun YX. STAT5 promotes PD-L1 expression by facilitating histone lactylation to drive immunosuppression in acute myeloid leukemia. Signal Transduct Target Ther. (2023) 8:391. doi: 10.1038/s41392-023-01605-2 37777506 PMC10542808

[96] Gu J Zhou J Chen Q Xu X Gao J Li X. Tumor metabolite lactate promotes tumorigenesis by modulating MOESIN lactylation and enhancing TGF-β signaling in regulatory T cells. Cell Rep. (2022) 39:110986. doi: 10.1016/j.celrep.2022.110986 35732125

[97] Yue Y Ren Y Lu C Li P Zhang G. Epigenetic regulation of human FOXP3+ Tregs: from homeostasis maintenance to pathogen defense. Front Immunol. (2024) 15:1444533. doi: 10.3389/fimmu.2024.1444533 39144146 PMC11323565

[98] Zhu Y Liu W Luo Z Xiao F Sun B. New insights into the roles of lactylation in cancer. Front Pharmacol. (2024) 15:1412672. doi: 10.3389/fphar.2024.1412672 39502530 PMC11534861

[99] Zhang X Liang C Wu C Wan S Xu L Wang S. A rising star involved in tumour immunity: Lactylation. J Cell Mol Med. (2024) 28:e70146. doi: 10.1111/jcmm.v28.20 39417674 PMC11483924

[100] Bao C Ma Q Ying X Wang F Hou Y Wang D. Histone lactylation in macrophage biology and disease: from plasticity regulation to therapeutic implications. EBioMedicine. (2024) 111:105502. doi: 10.1016/j.ebiom.2024.105502 39662177 PMC11697715

[101] Xiong J He J Zhu J Pan J Liao W Ye H. Lactylation-driven METTL3-mediated RNA m(6)A modification promotes immunosuppression of tumor-infiltrating myeloid cells. Mol Cell. (2022) 82:1660–77.e10. doi: 10.1016/j.molcel.2022.02.033 35320754

[102] Liao S Wu G Xie Z Lei X Yang X Huang S. pH regulators and their inhibitors in tumor microenvironment. Eur J Med Chem. (2024) 267:116170. doi: 10.1016/j.ejmech.2024.116170 38308950

[103] Pilon-Thomas S Kodumudi KN El-Kenawi AE Russell S Weber AM Luddy K. Neutralization of tumor acidity improves antitumor responses to immunotherapy. Cancer Res. (2016) 76:1381–90. doi: 10.1158/0008-5472.CAN-15-1743 PMC4829106 26719539

[104] Hosonuma M Yoshimura K. Association between pH regulation of the tumor microenvironment and immunological state. Front Oncol. (2023) 13:1175563. doi: 10.3389/fonc.2023.1175563 37492477 PMC10363976

[105] Rahman A Janic B Rahman T Singh H Ali H Rattan R. Immunotherapy enhancement by targeting extracellular tumor pH in triple-negative breast cancer mouse model. Cancers (Basel). (2023) 15(20):4931. doi: 10.3390/cancers15204931 37894298 PMC10605606

[106] Bogdanov A Bogdanov A Chubenko V Volkov N Moiseenko F Moiseyenko V. Tumor acidity: From hallmark of cancer to target of treatment. Front Oncol. (2022) 12:979154. doi: 10.3389/fonc.2022.979154 36106097 PMC9467452

[107] Wang BY Zhang J Wang JL Sun S Wang ZH Wang LP. Intermittent high dose proton pump inhibitor enhances the antitumor effects of chemotherapy in metastatic breast cancer. J Exp Clin Cancer Res. (2015) 34:85. doi: 10.1186/s13046-015-0194-x 26297142 PMC4546346

[108] Zhang JL Liu M Yang Q Lin SY Shan HB Wang HY. Effects of omeprazole in improving concurrent chemoradiotherapy efficacy in rectal cancer. World J Gastroenterol. (2017) 23:2575–84. doi: 10.3748/wjg.v23.i14.2575 PMC5394521 28465642

[109] Hebert KA Jaramillo S Yu W Wang M Veeramachaneni R Sandulache VC. Esomeprazole enhances the effect of ionizing radiation to improve tumor control. Oncotarget. (2021) 12. doi: 10.18632/oncotarget.v12i14 PMC8274720 34262645

[110] Hopkins AM Kichenadasse G McKinnon RA Abuhelwa AY Logan JM Badaoui S. Efficacy of first-line atezolizumab combination therapy in patients with non-small cell lung cancer receiving proton pump inhibitors: *post hoc* analysis of IMpower150. Br J Cancer. (2022) 126:42–7. doi: 10.1038/s41416-021-01606-4 PMC8727569 34711947

[111] Okamoto K Saito Y Yamaguchi A Takekuma Y Sugawara M. Acid suppressants reduce the therapeutic effect of immune checkpoint inhibitors and increase the risk of acute kidney injury: a meta-analysis. Int J Clin Oncol. (2023) 28:1343–53. doi: 10.1007/s10147-023-02385-z 37421477

[112] Nordfors K Haapasalo J Korja M Niemelä A Laine J Parkkila AK. The tumour-associated carbonic anhydrases CA II, CA IX and CA XII in a group of medulloblastomas and supratentorial primitive neuroectodermal tumours: an association of CA IX with poor prognosis. BMC Cancer. (2010) 10:148. doi: 10.1186/1471-2407-10-148 20398423 PMC2874782

[113] Ilie MI Hofman V Ortholan C Ammadi RE Bonnetaud C Havet K. Overexpression of carbonic anhydrase XII in tissues from resectable non-small cell lung cancers is a biomarker of good prognosis. Int J Cancer. (2011) 128:1614–23. doi: 10.1002/ijc.v128.7 20521252

[114] Tafreshi NK Lloyd MC Proemsey JB Bui MM Kim J Gillies RJ. Evaluation of CAIX and CAXII expression in breast cancer at varied O2 levels: CAIX is the superior surrogate imaging biomarker of tumor hypoxia. Mol Imaging Biol. (2016) 18:219–31. doi: 10.1007/s11307-015-0885-x PMC4754166 26276155

[115] Proescholdt MA Merrill MJ Stoerr EM Lohmeier A Pohl F Brawanski A. Function of carbonic anhydrase IX in glioblastoma multiforme. Neuro Oncol. (2012) 14:1357–66. doi: 10.1093/neuonc/nos216 PMC3480266 23074198

[116] Cetin B Gonul II Gumusay O Bilgetekin I Algin E Ozet A. Carbonic anhydrase IX is a prognostic biomarker in glioblastoma multiforme. Neuropathology. (2018) 38:457–62. doi: 10.1111/neup.2018.38.issue-5 29952031

[117] McDonald PC Chia S Bedard PL Chu Q Lyle M Tang L. A phase 1 study of SLC-0111, a novel inhibitor of carbonic anhydrase IX, in patients with advanced solid tumors. Am J Clin Oncol. (2020) 43:484–90. doi: 10.1097/COC.0000000000000691 PMC7323835 32251122

[118] Paškevičiūtė M Petrikaitė V. Application of carbonic anhydrase inhibitors to increase the penetration of doxorubicin and its liposomal formulation into 2D and 3D triple negative breast cancer cell cultures. Am J Cancer Res. (2020) 10:1761–9. PMC7339283 32642288

[119] Chen M Hu J Wang L Li Y Zhu C Chen C. Targeted and redox-responsive drug delivery systems based on carbonic anhydrase IX-decorated mesoporous silica nanoparticles for cancer therapy. Sci Rep. (2020) 10:14447. doi: 10.1038/s41598-020-71071-1 32879359 PMC7467921

[120] Boedtkjer E Pedersen SF. The acidic tumor microenvironment as a driver of cancer. Annu Rev Physiol. (2020) 82:103–26. doi: 10.1146/annurev-physiol-021119-034627 31730395

[121] Benjamin D Robay D Hindupur SK Pohlmann J Colombi M El-Shemerly MY. Dual inhibition of the lactate transporters MCT1 and MCT4 is synthetic lethal with metformin due to NAD+ depletion in cancer cells. Cell Rep. (2018) 25:3047–58.e4. doi: 10.1016/j.celrep.2018.11.043 30540938 PMC6302548

[122] Curtis NJ Mooney L Hopcroft L Michopoulos F Whalley N Zhong H. Pre-clinical pharmacology of AZD3965, a selective inhibitor of MCT1: DLBCL, NHL and Burkitt’s lymphoma anti-tumor activity. Oncotarget. (2017) 8:69219–36. doi: 10.18632/oncotarget.18215 PMC5642474 29050199

[123] Halford S Veal GJ Wedge SR Payne GS Bacon CM Sloan P. A phase I dose-escalation study of AZD3965, an oral monocarboxylate transporter 1 inhibitor, in patients with advanced cancer. Clin Cancer Res. (2023) 29:1429–39. doi: 10.1158/1078-0432.CCR-22-2263 PMC7614436 36652553

[124] Höckel M Vaupel P. Tumor hypoxia: definitions and current clinical, biologic, and molecular aspects. J Natl Cancer Inst. (2001) 93:266–76. doi: 10.1093/jnci/93.4.266 11181773

[125] Wang ZH Peng WB Zhang P Yang XP Zhou Q. Lactate in the tumour microenvironment: From immune modulation to therapy. EBioMedicine. (2021) 73:103627. doi: 10.1016/j.ebiom.2021.103627 34656878 PMC8524104

[126] Renner K Bruss C Schnell A Koehl G Becker HM Fante M. Restricting glycolysis preserves T cell effector functions and augments checkpoint therapy. Cell Rep. (2019) 29:135–50.e9. doi: 10.1016/j.celrep.2019.08.068 31577944

[127] Babl N Decking SM Voll F Althammer M Sala-Hojman A Ferretti R. MCT4 blockade increases the efficacy of immune checkpoint blockade. J Immunother Cancer. (2023) 11. doi: 10.1136/jitc-2023-007349 PMC10603342 37880183

[128] Gurrapu S Jonnalagadda SK Alam MA Nelson GL Sneve MG Drewes LR. Monocarboxylate transporter 1 inhibitors as potential anticancer agents. ACS Med Chem Lett. (2015) 6:558–61. doi: 10.1021/acsmedchemlett.5b00049 PMC4434469 26005533

[129] Benjamin D Hall MN. Combining metformin with lactate transport inhibitors as a treatment modality for cancer - recommendation proposal. Front Oncol. (2022) 12:1034397. doi: 10.3389/fonc.2022.1034397 36353534 PMC9637960

[130] Yao Y Zhou Y Liu L Xu Y Chen Q Wang Y. Nanoparticle-based drug delivery in cancer therapy and its role in overcoming drug resistance. Front Mol Biosci. (2020) 7:193. doi: 10.3389/fmolb.2020.00193 32974385 PMC7468194

[131] Hong L Li W Li Y Yin S. Nanoparticle-based drug delivery systems targeting cancer cell surfaces. RSC Adv. (2023) 13:21365–82. doi: 10.1039/D3RA02969G PMC10350659 37465582

[132] Mishra D Banerjee D. Lactate dehydrogenases as metabolic links between tumor and stroma in the tumor microenvironment. Cancers (Basel). (2019) 11. doi: 10.3390/cancers11060750 PMC6627402 31146503

[133] Verma S Budhu S Serganova I Dong L Mangarin LM Khan JF. Pharmacologic LDH inhibition redirects intratumoral glucose uptake and improves antitumor immunity in solid tumor models. J Clin Invest. (2024) 134(6):750. doi: 10.1172/JCI177606 PMC11364391 39225102

[134] Tang Y Gu S Zhu L Wu Y Zhang W Zhao C. LDHA: The Obstacle to T cell responses against tumor. Front Oncol. (2022) 12. doi: 10.3389/fonc.2022.1036477 PMC9742379 36518315

[135] Miao P Sheng S Sun X Liu J Huang G. Lactate dehydrogenase A in cancer: a promising target for diagnosis and therapy. IUBMB Life. (2013) 65:904–10. doi: 10.1002/iub.1216 24265197

[136] Coronel-Hernández J Salgado-García R Cantú-De-León D Jacobo-Herrera N Millan-Catalan O Delgado-Waldo I. Combination of metformin, sodium oxamate and doxorubicin induces apoptosis and autophagy in colorectal cancer cells via downregulation HIF-1α. Front Oncol. (2021) 11:594200. doi: 10.3389/fonc.2021.594200 34123772 PMC8187873

[137] Lu Q-Y Zhang L Yee JK Go V-LW Lee W-N. Metabolic consequences of LDHA inhibition by epigallocatechin gallate and oxamate in MIA PaCa-2 pancreatic cancer cells. Metabolomics. (2015) 11:71–80. doi: 10.1007/s11306-014-0672-8 26246802 PMC4523095

[138] Zhao Z Han F Yang S Wu J Zhan W. Oxamate-mediated inhibition of lactate dehydrogenase induces protective autophagy in gastric cancer cells: Involvement of the Akt–mTOR signaling pathway. Cancer letters. (2015) 358:17–26. doi: 10.1016/j.canlet.2014.11.046 25524555

[139] Yang Y Su D Zhao L Zhang D Xu J Wan J. Different effects of LDH-A inhibition by oxamate in non-small cell lung cancer cells. Oncotarget. (2014) 5:11886. doi: 10.18632/oncotarget.v5i23 25361010 PMC4323009

[140] Zhai X Yang Y Wan J Zhu R Wu Y. Inhibition of LDH-A by oxamate induces G2/M arrest, apoptosis and increases radiosensitivity in nasopharyngeal carcinoma cells. Oncol Rep. (2013) 30:2983–91. doi: 10.3892/or.2013.2735 24064966

[141] Le A Cooper CR Gouw AM Dinavahi R Maitra A Deck LM. Inhibition of lactate dehydrogenase A induces oxidative stress and inhibits tumor progression. Proc Natl Acad Sci U S A. (2010) 107:2037–42. doi: 10.1073/pnas.0914433107 PMC2836706 20133848

[142] Rajeshkumar NV Dutta P Yabuuchi S de Wilde RF Martinez GV Le A. Therapeutic targeting of the warburg effect in pancreatic cancer relies on an absence of p53 function. Cancer Res. (2015) 75:3355–64. doi: 10.1158/0008-5472.CAN-15-0108 PMC4537812 26113084

[143] Guo L Yang Y Sheng Y Wang J Li W Zhou X. Galloflavin relieves the Malignant behavior of colorectal cancer cells in the inflammatory tumor microenvironment. Front Pharmacol. (2021) 12:752118. doi: 10.3389/fphar.2021.752118 34955826 PMC8702829

[144] Han X Sheng X Jones HM Jackson AL Kilgore J Stine JE. Evaluation of the anti-tumor effects of lactate dehydrogenase inhibitor galloflavin in endometrial cancer cells. J Hematol Oncol. (2015) 8:1–4. doi: 10.1186/s13045-014-0097-x 25631326 PMC4316809

[145] Farabegoli F Vettraino M Manerba M Fiume L Roberti M Di Stefano G. Galloflavin, a new lactate dehydrogenase inhibitor, induces the death of human breast cancer cells with different glycolytic attitude by affecting distinct signaling pathways. Eur J Pharm Sci. (2012) 47:729–38. doi: 10.1016/j.ejps.2012.08.012 22954722

[146] Sharma D Singh M Rani R. Role of LDH in tumor glycolysis: Regulation of LDHA by small molecules for cancer therapeutics. Semin Cancer Biol. (2022) 87:184–95. doi: 10.1016/j.semcancer.2022.11.007 36371026

[147] Chaube B Malvi P Singh SV Mohammad N Meena AS Bhat MK. Targeting metabolic flexibility by simultaneously inhibiting respiratory complex I and lactate generation retards melanoma progression. Oncotarget. (2015) 6:37281–99. doi: 10.18632/oncotarget.v6i35 PMC4741930 26484566

[148] Shibata S Sogabe S Miwa M Fujimoto T Takakura N Naotsuka A. Identification of the first highly selective inhibitor of human lactate dehydrogenase B. Sci Rep. (2021) 11:21353. doi: 10.1038/s41598-021-00820-7 34725423 PMC8560939

[149] Rai G Urban DJ Mott BT Hu X Yang SM Benavides GA. Pyrazole-based lactate dehydrogenase inhibitors with optimized cell activity and pharmacokinetic properties. J Med Chem. (2020) 63:10984–1011. doi: 10.1021/acs.jmedchem.0c00916 PMC7830743 32902275

[150] Sheppard S Santosa EK Lau CM Violante S Giovanelli P Kim H. Lactate dehydrogenase A-dependent aerobic glycolysis promotes natural killer cell anti-viral and anti-tumor function. Cell Rep. (2021) 35:109210. doi: 10.1016/j.celrep.2021.109210 34077737 PMC8221253

[151] Ma J Tang L Tan Y Xiao J Wei K Zhang X. Lithium carbonate revitalizes tumor-reactive CD8(+) T cells by shunting lactic acid into mitochondria. Nat Immunol. (2024) 25:552–61. doi: 10.1038/s41590-023-01738-0 PMC10907288 38263463

[152] Chaube B Bhat MK. AMPK, a key regulator of metabolic/energy homeostasis and mitochondrial biogenesis in cancer cells. Cell Death Dis. (2016) 7:e2044. doi: 10.1038/cddis.2015.404 26775698 PMC4816182

[153] Xu Y Ma K Zhang L Li G. Supercharging cancer-fighting T cells with lithium carbonate. Cell Metab. (2024) 36:463–5. doi: 10.1016/j.cmet.2024.02.006 38447529

[154] Walenta S Chau TV Schroeder T Lehr HA Kunz-Schughart LA Fuerst A. Metabolic classification of human rectal adenocarcinomas: a novel guideline for clinical oncologists? J Cancer Res Clin Oncol. (2003) 129(6):321–6. doi: 10.1007/s00432-003-0450-x PMC12161967 12827509

[155] Vlachostergios PJ Oikonomou KG Gibilaro E Apergis G. Elevated lactic acid is a negative prognostic factor in metastatic lung cancer. Cancer biomark. (2015) 15:725–34. doi: 10.3233/CBM-150514 PMC12965472 26406401

[156] Nikoobakht M Shamshiripour P Azimi Nekoo Z Fallah Haghmohammadi S. Elevated lactate and total protein levels in stereotactic brain biopsy specimen; potential biomarkers of Malignancy and poor prognosis. Arch Iran Med. (2019) 22:125–31. 31029068

[157] Sun RC Fadia M Dahlstrom JE Parish CR Board PG Blackburn AC. Reversal of the glycolytic phenotype by dichloroacetate inhibits metastatic breast cancer cell growth *in vitro* and *in vivo* . Breast Cancer Res Treat. (2010) 120:253–60. doi: 10.1007/s10549-009-0435-9 19543830

[158] Cao W Yacoub S Shiverick KT Namiki K Sakai Y Porvasnik S. Dichloroacetate (DCA) sensitizes both wild-type and over expressing Bcl-2 prostate cancer cells *in vitro* to radiation. Prostate. (2008) 68:1223–31. doi: 10.1002/pros.v68:11 18465755

[159] Madhok BM Yeluri S Perry SL Hughes TA Jayne DG. Dichloroacetate induces apoptosis and cell-cycle arrest in colorectal cancer cells. Br J Cancer. (2010) 102:1746–52. doi: 10.1038/sj.bjc.6605701 PMC2883702 20485289

[160] Michelakis ED Sutendra G Dromparis P Webster L Haromy A Niven E. Metabolic modulation of glioblastoma with dichloroacetate. Sci Transl Med. (2010) 2:31ra4. doi: 10.1126/scitranslmed.3000677 20463368

[161] Herst PM Howman RA Neeson PJ Berridge MV Ritchie DS. The level of glycolytic metabolism in acute myeloid leukemia blasts at diagnosis is prognostic for clinical outcome. J Leukoc Biol. (2011) 89:51–5. doi: 10.1189/jlb.0710417 20959411

[162] Sanchez WY McGee SL Connor T Mottram B Wilkinson A Whitehead JP. Dichloroacetate inhibits aerobic glycolysis in multiple myeloma cells and increases sensitivity to bortezomib. Br J Cancer. (2013) 108:1624–33. doi: 10.1038/bjc.2013.120 PMC3668464 23531700

[163] Flavin DF. Non-hodgkin’s lymphoma reversal with dichloroacetate. J Oncol. (2010) 2010:414726. doi: 10.1155/2010/414726 20886020 PMC2945664

[164] Kolesnik DL Pyaskovskaya ON Boychuk IV Dasyukevich OI Melnikov OR Tarasov AS. Effect of dichloroacetate on Lewis lung carcinoma growth and metastasis. Exp Oncol. (2015) 37:126–9. doi: 10.31768/2312-8852.2015.37(2):126-129 26112940

[165] Young MJ Wang SA Chen YC Liu CY Hsu KC Tang SW. USP24-i-101 targeting of USP24 activates autophagy to inhibit drug resistance acquired during cancer therapy. Cell Death Differ. (2024) 31:574–91. doi: 10.1038/s41418-024-01277-7 PMC11093971 38491202

[166] Giambra M Di Cristofori A Raimondo F Rigolio R Conconi D Chiarello G. Vacuolar proton-translocating ATPase may take part in the drug resistance phenotype of glioma stem cells. Int J Mol Sci. (2024) 25(5):2743. doi: 10.3390/ijms25052743 38473989 PMC10932054

[167] Koltai T Fliegel L. Dichloroacetate for cancer treatment: some facts and many doubts. Pharm (Basel). (2024) 17(6):744. doi: 10.3390/ph17060744 PMC11206832 38931411

[168] Li B Liu Y Sun S. Proton pump inhibitors display anti-tumour potential in glioma. Cell Prolif. (2023) 56:e13321. doi: 10.1111/cpr.13321 35961680 PMC10334276

[169] Gezmiş T Albayrak K Parlak SN Ulusoy D Yilmaz AT. Investigation of the effects of omeprazole, a proton pump inhibitor, on apoptotic gene regions on human fibroblast cells. Eurasian J Mol Biochem Sci. (2024) 3:10–6.

[170] Li M Zeng C Yao J Ge Y An G. The association between proton pump inhibitors use and clinical outcome of patients receiving immune checkpoint inhibitors therapy. Int Immunopharmacol. (2020) 88:106972. doi: 10.1016/j.intimp.2020.106972 33182025

[171] Dar S Merza N Qatani A Rahim M Varughese T Mohammad A. Impact of proton-pump inhibitors on the efficacy of immune checkpoint inhibitors in non-small cell lung cancer: A systematic review and meta-analysis. Ann Med Surg (Lond). (2022) 78:103752. doi: 10.1016/j.amsu.2022.103752 35600176 PMC9119820

[172] Qin BD Jiao XD Zhou XC Shi B Wang J Liu K. Effects of concomitant proton pump inhibitor use on immune checkpoint inhibitor efficacy among patients with advanced cancer. Oncoimmunology. (2021) 10:1929727. doi: 10.1080/2162402X.2021.1929727 34350061 PMC8296970

[173] Clemmons AB Chase A Duong P Waller JL Saunders K Bryan L. Assessing the impact of adding acetazolamide to oral or intravenous sodium bicarbonate as compared with intravenous bicarbonate monotherapy as urinary alkalinization in adults receiving high-dose methotrexate. Support Care Cancer. (2021) 29:1527–34. doi: 10.1007/s00520-020-05646-z 32725375

[174] Gillies RJ Ibrahim-Hashim A Ordway B Gatenby RA. Back to basic: Trials and tribulations of alkalizing agents in cancer. Front Oncol. (2022) 12:981718. doi: 10.3389/fonc.2022.981718 36452492 PMC9702334

[175] Heuser C Renner K Kreutz M Gattinoni L. Targeting lactate metabolism for cancer immunotherapy - a matter of precision. Semin Cancer Biol. (2023) 88:32–45. doi: 10.1016/j.semcancer.2022.12.001 36496155

[176] Miranda-Gonçalves V Honavar M Pinheiro C Martinho O Pires MM Pinheiro C. Monocarboxylate transporters (MCTs) in gliomas: expression and exploitation as therapeutic targets. Neuro Oncol. (2013) 15:172–88. doi: 10.1093/neuonc/nos298 PMC3548586 23258846

[177] Morais-Santos F Miranda-Gonçalves V Pinheiro S Vieira AF Paredes J Schmitt FC. Differential sensitivities to lactate transport inhibitors of breast cancer cell lines. Endocr Relat Cancer. (2014) 21:27–38. doi: 10.1530/ERC-13-0132 24174370

[178] Kong SC Nøhr-Nielsen A Zeeberg K Reshkin SJ Hoffmann EK Novak I. Monocarboxylate transporters MCT1 and MCT4 regulate migration and invasion of pancreatic ductal adenocarcinoma cells. Pancreas. (2016) 45:1036–47. doi: 10.1097/MPA.0000000000000571 26765963

[179] Jonnalagadda S Jonnalagadda SK Ronayne CT Nelson GL Solano LN Rumbley J. N-dialkyl cyanocinnamic acids as monocarboxylate transporter 1 and 4 inhibitors. Oncotarget. (2019) 10:2355. doi: 10.18632/oncotarget.v10i24 31040927 PMC6481325

[180] Sandforth L Ammar N Dinges LA Röcken C Arlt A Sebens S. Impact of the monocarboxylate transporter-1 (MCT1)-mediated cellular import of lactate on stemness properties of human pancreatic adenocarcinoma cells †. Cancers (Basel). (2020) 12(3):581. doi: 10.3390/cancers12030581 32138176 PMC7139999

[181] Guan X Rodriguez-Cruz V Morris ME. Cellular uptake of MCT1 inhibitors AR-C155858 and AZD3965 and their effects on MCT-mediated transport of L-lactate in murine 4T1 breast tumor cancer cells. AAPS J. (2019) 21:1–10. doi: 10.1208/s12248-018-0279-5 PMC6466617 30617815

[182] Vander Linden C Corbet C Bastien E Martherus R Guilbaud C Petit L. Therapy-induced DNA methylation inactivates MCT1 and renders tumor cells vulnerable to MCT4 inhibition. Cell Rep. (2021) 35:109202. doi: 10.1016/j.celrep.2021.109202 34077729

[183] Kim EY Chung TW Han CW Park SY Park KH Jang SB. A novel lactate dehydrogenase inhibitor, 1-(Phenylseleno)-4-(Trifluoromethyl) benzene, suppresses tumor growth through apoptotic cell death. Sci Rep. (2019) 9:3969. doi: 10.1038/s41598-019-40617-3 30850682 PMC6408513

[184] Qi C-L Huang M-L Zou Y Yang R Jiang Y Sheng J-F. The IRF2/CENP-N/AKT signaling axis promotes proliferation, cell cycling and apoptosis resistance in nasopharyngeal carcinoma cells by increasing aerobic glycolysis. J Exp Clin Cancer Res. (2021) 40:390. doi: 10.1186/s13046-021-02191-3 34893086 PMC8662847

[185] Gupta VK Sharma NS Durden B Garrido VT Kesh K Edwards D. Hypoxia-driven oncometabolite L-2HG maintains stemness-differentiation balance and facilitates immune evasion in pancreatic cancer. Cancer Res. (2021) 81:4001–13. doi: 10.1158/0008-5472.CAN-20-2562 PMC8338764 33990397

[186] Meléndez AV Velasco Cárdenas RM-H Lagies S Strietz J Siukstaite L Thomas OS. Novel lectin-based chimeric antigen receptors target Gb3-positive tumour cells. Cell Mol Life Sci. (2022) 79:513. doi: 10.1007/s00018-022-04524-7 36097202 PMC9468074

[187] Schwab M Thunborg K Azimzadeh O von Toerne C Werner C Shevtsov M. Targeting cancer metabolism breaks radioresistance by impairing the stress response. Cancers. (2021) 13:3762. doi: 10.3390/cancers13153762 34359663 PMC8345170

[188] Varma G Seth P Coutinho de Souza P Callahan C Pinto J Vaidya M. Visualizing the effects of lactate dehydrogenase (LDH) inhibition and LDH-A genetic ablation in breast and lung cancer with hyperpolarized pyruvate NMR. NMR Biomedicine. (2021) 34:e4560. doi: 10.1002/nbm.v34.8 PMC8764798 34086382

[189] Maeda M Ko M Mane MM Cohen IJ Shindo M Vemuri K. Genetic and drug inhibition of LDH-A: Effects on murine gliomas. Cancers. (2022) 14:2306. doi: 10.3390/cancers14092306 35565435 PMC9105502

[190] Zhou Y Tao P Wang M Xu P Lu W Lei P. Development of novel human lactate dehydrogenase A inhibitors: High-throughput screening, synthesis, and biological evaluations. Eur J Med Chem. (2019) 177:105–15. doi: 10.1016/j.ejmech.2019.05.033 31129449

[191] Fang A Zhang Q Fan H Zhou Y Yao Y Zhang Y. Discovery of human lactate dehydrogenase A (LDHA) inhibitors as anticancer agents to inhibit the proliferation of MG-63 osteosarcoma cells. Medchemcomm. (2017) 8:1720–6. doi: 10.1039/C7MD00222J PMC6072013 30108883

